# Quiescence: early evolutionary origins and universality do not imply uniformity

**DOI:** 10.1098/rstb.2011.0079

**Published:** 2011-12-27

**Authors:** Patrick H. O'Farrell

**Affiliations:** Department of Biochemistry, University of California, San Francisco, CA 94158-2200, USA

**Keywords:** quiescence, growth, size, evolution

## Abstract

Cell cycle investigations have focused on relentless exponential proliferation of cells, an unsustainable situation in nature. Proliferation of cells, whether microbial or metazoan, is interrupted by periods of quiescence. The vast majority of cells in an adult metazoan lie quiescent. As disruptions in this quiescence are at the foundation of cancer, it will be important for the field to turn its attention to the mechanisms regulating quiescence. While often presented as a single topic, there are multiple forms of quiescence each with complex inputs, some of which are tied to conceptually challenging aspects of metazoan regulation such as size control. In an effort to expose the enormity of the challenge, I describe the differing biological purposes of quiescence, and the coupling of quiescence in metazoans to growth and to the structuring of tissues during development. I emphasize studies in the organism rather than in tissue culture, because these expose the diversity of regulation. While quiescence is likely to be a primitive biological process, it appears that in adapting quiescence to its many distinct biological settings, evolution has diversified it. Consideration of quiescence in different models gives us an overview of this diversity.

## 1. Introduction

Most cells of an adult metazoan have exited the cell cycle and generally lie quiescent unless called upon to replace cells lost to injury or turnover, or unless an oncogenic change disrupts the quiescence and drives pathological proliferation. This quiescence is central to normal metazoan biology and its disruptions underlie cancer. However, the cell cycle field has largely focused on experimental systems exhibiting unchecked growth and proliferation. It is increasingly recognized that an understanding of cell cycle regulation in its normal context will benefit from a focus on the regulation of quiescence and its disruptions. But, I think the magnitude of this question is not widely appreciated. Here, I suggest that it is a huge biological issue that will interface tightly with development, as well as nutrition, evolution and cancer biology. In this respect, research into this area appears to be poised for major expansion. I highlight the expanse of the topic by focusing on the complexity of quiescence in the metazoan context and I consider the optimistic perspective that quiescence, as practised in various simple models, will define a global mechanism that is relevant from yeast to mammals.

The term quiescence has been used to cover a broad range of circumstances, which obfuscates comparisons between organisms. There are numerous biological uses for quiescence, as well as different forms of it. I outline some of each of these as groundwork for a discussion of the possible connections between the quiescence practised in different organisms. We will see that organisms possess multiple forms of quiescence and selectively engage these at different times to achieve outcomes suited to their lifestyle.

Rapidly growing cells have much in common, as they all actively express growth genes, such as genes encoding machinery that makes more protein ([1,2]; O'Farrell 1976, unpublised data). In contrast, quiescent cells are free to adopt any specialization and can differ dramatically [2,3]. Indeed, the major defining feature of the ‘state of quiescence’ is that quiescent cells do not engage in one or more activities practised by non-quiescent cells. Additionally, there is a diversity of signals that trigger quiescence in different systems. If there are global generalities to be made, then they are likely to appear in the mechanisms that are called forth to shut down activities associated with growth.

## 2. The origins of quiescence

There are strong arguments favouring the notion that quiescence makes integral contributions to biological success and it is likely to have evolved early. It has long been recognized that successful exponential growth will inevitably surmount the supplies needed to support it. It was recognition of this tension that motivated Thomas Malthus's influential ‘An Essay on the Principle of Population’, which starkly presented the challenges of human population growth for society. However, the tension is universal, and was recognized by Malthus who wrote ‘The cause to which I allude, is the constant tendency in all animated life to increase beyond the nourishment prepared for it’ [4, p. 2]. This tension impacts all life forms and it underlies evolutionary selection for mechanisms to survive the limitation that is inevitable.

A little mental exercise illustrates the tension between growth and resources in a prokaryotic context. I do not remember where I first heard about this simple calculation, but I have confirmed it myself, and I never cease to find it impressive [5]. A single *Escherichia coli* weighs about 10^−12^ g [6]. If well fed, it will double every 20 min and conveniently grows quickly to numbers adequate for experimental purposes. But, consider the consequences of three doublings per hour if you could keep *E. coli* well fed for just a little longer than our usual cultures. In 24 h (72 doublings), you would have 4000 metric tonnes of *E. coli* (10^−12^ g × 2^72^). In 2 days, the mass of *E. coli* (∼1.6 × 10^28^ kg) would be larger than the mass of the Earth (∼6 × 10^24^ kg) and in 3 days this mass would have grown to occupy a volume (at ∼1 g cm^−3^ this = ∼6.4 × 10^46^ m^3^ ≈ 2 × 10^13^ AU^3^ ≈ 6 × 10^−2^ cubic light years) larger than the solar system (∼2.7 × 10^5^ AU^3^, assuming a sphere enclosing the planetary disc), with boundaries expanding through the universe at a greater speed than the speed of light. This somewhat silly mathematical exercise illustrates the awesome potential of exponential growth. Clearly, it is unsustainable, and the implications are general. Although a 20 min doubling time is exceptionally fast, the 3 days encompassed by this calculation are a small part of the approximately 3.6 Gyr of life on the Earth. Thus, the available resources, as Malthus pointed out, will eventually limit the growth of even slowly growing organisms. Micro-organisms benefit from an ability to survive periods of limitation in quiescence if they then can emerge from this quiescence and flourish when conditions are again favourable. This advantage creates a powerful evolutionary drive for effective forms of quiescence.

If quiescence appeared early in evolution, then this capability might have been passed on and so spread widely in phylogeny. Consistent with this, we find well-developed nutritional control of growth and proliferation in diverse organisms. Although spread of a primordial regulatory scheme could result in a common regulatory mechanism used throughout phylogeny, evolutionary specialization could diversify the initial mechanism or add new mechanisms. Indeed, diversity is evident in the natural histories of many organisms that feature specialized quiescent zygotes or spores that are the centrepiece of varied reproductive strategies emphasizing survival and dispersal. Apparently, quiescence has been adapted to suit the specialized purposes of diverse biological contexts.

## 3. Distinctions in the biological purpose of quiescence

In the earlier-mentioned discussion, I have conflated two forms of quiescence having different purposes. One type of quiescence is induced by deprivation and it serves the purpose of aiding the survival of cells until more opportune times. But the cells of an adult mammal are housed in a protected nutritive environment. What is the purpose of their quiescence and what is the inducing signal? Quiescence in the mammal serves the purpose of the entire organism rather than the individual cells. One purpose of quiescence is to arrest the growth of the organism. Stopping growth at an appropriate adult size has been selected because metazoans have complex body plans that function optimally at a particular size. Additionally, arrest of proliferation has the advantage that it releases constraints on differentiation in that it frees cells to adopt specializations that might impede cell division [7].

While some metazoans also exhibit a developmental quiescence, a diapause, in response to nutritional deprivation, I will not deal with such diapauses here.

It is not immediately apparent how distinctions in biological purpose might impact on the mechanisms used, except that one can assume that the inducers of quiescence vary depending on the purpose. The issue of the inducing mechanism is particularly mysterious and complex in metazoans. In an attempt to build a foundation for comparing quiescence in different systems, I begin with considerations of the conditions and signals that induce quiescence in metazoans.

## 4. Size control and coupling to quiescence

Animals vary enormously in size [7]. Just among mammals, the range is about 10^7^-fold from a 15 g mouse to a 150 tonnes blue whale. But increasing scale requires a change in the body structure to accommodate the fact that attributes such as weight and limb strength do not maintain the same proportion as size increases—weight increases in proportion to the cube of linear dimensions, whereas limb strength increases in proportion to the square of linear dimensions. Thus, each body plan functions optimally only within a limited range of sizes. Although the mammalian embryo can grow many orders of magnitude *in utero* during foetal growth, mammals in every size range grow roughly 20-fold from birth to maturity, at which time growth ceases [7]. From this, it appears that physical constraints dictate a widespread developmental pattern that limits body size to a narrow range compatible with function. Notably, cessation of growth of the organism involves cessation of cellular growth and proliferation. Thus, the slowing of growth is achieved by the induction of quiescence.

The control of size is one of the most mysterious aspects of biology. Its influence is also widely under-appreciated. Size is not only regulated at the end of growth, but it is also regulated throughout development. Indeed, the shaping of organs and the organism itself is owing to the regulation of the size of its parts. The near perfection of bilateral symmetry highlights the accuracy with which size regulation proportions different body parts. Although we understand little of the mechanisms of size regulation, increased interest and attention have led to several new discoveries and stimulated a number of reviews that outline the features of growth control and identify some of the inputs [7–12]. Here, I summarize some of the important insights, as these impinge on our understanding of the regulation of quiescence.

A coupling of quiescence with the approach to full size can be seen in numerous systems. In mammals, there is progressive slowing of growth rate as proliferation slows [10,13]. In *Drosophila*, size regulation is most clearly evident and most thoroughly studied in the imaginal discs, groups of larval cells that comprise the anlagen of adult structures. The wing disc cells grow and proliferate exponentially from the middle of the first larval instar throughout the larval period, slowing and ultimately arresting as the disc reaches full size [14].

A number of mutations alter growth so that all body parts increase in proportion [15,16]. This finding suggests that there are systemic signals that regulate the size of the organism. Indeed, endocrine factors, such as insulin-like growth factor, have been found to influence body size in flies as well as in mammals [10,15,17]. However, the levels of these hormones do not decline in parallel with growth, and animals deficient in their production still exhibit size control, albeit at a reduced size [10,15,17]. Thus, there clearly must be other factors contributing to the onset of growth quiescence with maturity.

Transplantation of tissues/organs in flies and mammals shows that organ- or tissue-autonomous signals play an important role in size control [7–12]. For example, in mammals, transplantation of foetal thymus glands into an adult is followed by the growth of the tissue to the normal full size, which is followed by cessation of growth [11]. If the full-sized wing disc is offered further opportunity to grow and proliferate by delaying formation of the pupa, or by transplanting the disc undamaged into a younger larva, then it remains quiescent [18]. Cutting the disc induces a regenerative response that reveals a continued capacity for growth and cell duplication [14]. Thus, the quiescence, which is induced only when the disc reaches full size, is intrinsic to the disc and requires that the disc remain intact. These observations show that final size triggers cellular quiescence.

Where described, growth of a metazoan does not progress exponentially to a sudden arrest at the size of the mature organism, but rather shows a gradual slowing as a juvenile approaches full size. This feature, along with the existence of both promoters and inhibitors of growth, suggests that growth is a graded phenomenon and that it integrates many inputs. Importantly, not all regulatory inputs that influence final size need change with growth. Perhaps only one input changes as an embryo/animal grows, whether an inhibitory input that increases with size or a growth promoting input that decreases with size. All the other inputs might be more or less constant or carry information about parameters other than size (e.g. nutrition) and could adjust the size threshold at which the balance of signals no longer promotes growth. Based on the autonomy of growth control in tissues and mutations that cause overgrowth in *Drosophila*, I suggest that tissue/organ-autonomous signals produce a growth inhibitory input that increases in magnitude as size increases [14,19]. Accordingly, systemic growth signals would operate on all tissues, and growth of each tissue would stall when it reached a size where the integrated inputs of local and systemic signals no longer promote growth. Such a model could account for the various nutritional and environmental inputs into growth and overall size [10,20,21]. It also argues that numerous signals are likely to be integrated by the circuits that regulate quiescence.

## 5. Diverse tissue-specific programmes of growth control

Growth and development proceed differently in different tissues. We can identify at least three general types of regulatory programmes. In one type of regulation, exemplified by tissues such as the fly discs and the mammalian liver, growth and proliferation are widespread, occurring throughout the tissue. This dispersed growth and proliferation slows to almost a complete stop at the growth limit. In a second pattern of growth and proliferation, there are specialized proliferative cells that can be concentrated in growth zones (e.g. the epiphyseal plates of bone) or dispersed throughout the tissue (e.g. the satellite cells of muscle). In a third pattern of growth and proliferation, every division appears to be developmentally programmed according to an exact schedule dictated by other events in development. Furthermore, as detailed below, individual tissues practising each of these styles of regulation can exhibit additional distinctions in their regulation of quiescence.

Tissues that exhibit widespread proliferation prior to quiescence (the first pattern of growth and proliferation) can arrest in different ways. For example, the arrest of larval proliferation in the *Drosophila* eye disc is associated with a specialized morphogenetic wave of differentiation that traverses the eye, whereas the cells of the wing disc, with the exception of a minor population of early arresting cells at the wing margin, behave relatively uniformly and gradually extend their cell cycle [14,22–25].

In tissues with specialized generative or stem cell populations (the second pattern of growth and proliferation), differentiation is associated with an exit from the cell cycle. This type of control of growth and proliferation is associated with multiple kinds of quiescence—the differentiating population of cells ceases proliferating (proliferation quiescence) but the differentiated cells can continue to grow in size until a separate onset of growth quiescence. Furthermore, the quiescence of the generative cells themselves is distinctly regulated.

Unlike the quiescence of tissues such as liver or the discs of the *Drosophila* larva, the timing of proliferation quiescence in systems with a specialized generative population of cells is coupled to differentiation and not closely coupled to attainment of final size. Differentiation-associated quiescence accompanies some of the first events of tissue differentiation in development and continues into adulthood where stem cells continue to replace particular tissues that turnover (e.g. skin), and where stem cells can be reactivated during the repair after wounding or injury.

The proliferation quiescence of differentiated cells is not necessarily associated with quiescence in cellular growth. For example, neurons often grow immensely. Following its formation in the embryo, the axon of a motor neuron innervating the leg of a giraffe will grow from micrometres to metres and do so in coordination with organismal growth. Thus, differentiation quiescence differs from growth quiescence in its features as well as in its schedule. Furthermore, when one considers the growth of a neuron, for example, it appears that in addition to the proliferation quiescence that is engaged when it first embarks on the pathway to differentiation, there has to be a second type of regulation that modulates growth, which ultimately leads to growth quiescence at maturity.

In addition to a proliferation quiescence and a growth quiescence associated with the differentiating cells, tissues with a generative population can exhibit one more type of quiescence—quiescence of the stem cells whose activity is an important driver of the growth. For example, overall stature is largely the result of the growth of long bones in juveniles. This growth is driven by the activity of the epiphyseal plate, a generative layer that deposits cartilage that is ossified as it is displaced from the plate. The epiphyseal plate matures and loses activity at puberty. Like the growth of the liver and the growth of the already formed axons, the activity of the epiphyseal plate is coupled to size and maturity. But as noted already, the activity of generative cells, such as the basal cells in the skin, crypt cells in the gut and glial stem cells, is not coupled to organismal size. Distinct developmental inputs appear to coordinate the activity of different stem cell populations.

The third style of growth and proliferation is highly regimented. For example, the proliferation that gives rise to the soma of the *Caenorhabditis elegans* worm follows an almost invariant lineage, and the few examples of a probabilistic cell fate specification appear to be genetically programmed switches. The early fly embryo has only slightly less rigidly stereotyped divisions. In the fly embryo, 13 synchronous cell cycles are followed by three cell cycles that follow position-dependent schedules. Almost all cells exit the mitotic cell cycle in cycle 16. Even though there is no precise lineage of the divisions within stereotyped territories, the division domains [26], the number and time of the all the divisions are specified by inputs from the patterning genes guiding early development. The spatially programmed divisions are limited by the availability of the mitotic phosphatase Cdc25 encoded by *string*, whose transcription is dependent on developmental regulators known to govern the morphological pattern of the embryo [8,27,28]. These developmental regulators are transcription factors or the regulators of transcription factors, and they are expressed in spatial and temporal patterns in the embryo. Like coordinates for latitude and longitude, combinations of these developmental regulators can specify position. Additionally, the levels of the regulators reflect time. Acting in combinations, they promote Cdc25 expression according to a spatial and temporal schedule that then drives the patterned mitoses [8]. These examples show that at times, developmental control acts as an exacting master continuously dictating the precise schedule of cell cycle progression. In such a context, these rigid developmental programmes also regulate the onset of quiescence (see later text).

These brief considerations of quiescence in metazoans emphasize its connection to development and introduce the notion that there are diverse forms of quiescence. The diverse manner in which quiescence is coupled to growth in different tissues and organs suggests that evolution has engaged varied types of tissue-specific developmental programmes to regulate quiescence.

## 6. From whence the diversity

Early evolution of quiescence and its widespread association with maturation in metazoans educe thoughts of universality, but the inputs into quiescence in metazoans display a bewildering diversity that challenges our ability to see generalities. With the exception of a few groupings of tissues with parallel developmental courses, patterns of growth and proliferation are remarkably tissue-specific. Despite the complexity, there is a gratifying appropriateness to the diversity, as the details of the growth programme for each tissue are beautifully tailored to the specific structures being produced. Indeed, it is what one expects for a process regulated as part of the developmental programme.

Evolution has produced extraordinarily diverse body shapes and sizes. This can be understood in part as the result of the fact that natural selection acts largely on the structure and performance of an organism's body. However, the rapid diversification requires plasticity in the mechanisms governing shape and size. A precedent suggests an origin of the plasticity. Upon recognizing that the development of each segment of the insect body plan can be distinct from any other segment because of the action of particular homeotic genes, E. B. Lewis suggested that the independence allowed each segment to be a separate experiment in evolution—that is, variants could affect the pattern of only one segment and those changes that were advantageous could be selected without compromising the development of the remaining segments [7,29]. Similarly, autonomous and distinct control of the size of individual body parts allows evolution to modify body structure by changes in the relative size of different parts—examples of which are the elephant's trunk, the giraffe's neck and the butterfly's wings.

It is clear that not every example of diversity in developmental programming marks a diversity of mechanistic inputs. The same regulators can be used in different developmental contexts. For example, the halteres of *Drosophila* are small vestigial wing-like structures on the third thoracic segment that can be transformed into wings if they lose the expression of the homeotic gene, *Ubx*. Ubx influences the size of these structures by altering the gradients of two morphogens, Wingless and Dpp (BMP homolog), whose expression in source cells is directed by Notch and Hedgehog, respectively [9]. More impressively, the same regulators can be used in structures that are not analogous and that use dissimilar morphogenetic processes. For example, the important signalling molecules Hedgehog, Wingless, Dpp and Notch are central players in controlling growth and proliferation in both the wing disc and eye disc, in which there is no obvious parallel between the programmes of cell cycle and growth arrest [14,30–33]. Apparently, changes in where and when they and their collaborating regulators are produced allow these conserved regulators to function as pivotal determinants of dissimilar programmes.

This discussion of quiescence emphasizes the specificity of programming in different tissues within the organism and suggests that this is because growth, proliferation and quiescence are inherent components of the developmental programme.

## 7. Quiescence of what?

Much of the literature on quiescence discusses the problem as one of how cells exit the cell cycle. As cells exit the cell cycle during quiescence, this is indeed part of the problem, but often quiescence involves more than this exit. To survive nutritional quiescence, micro-organisms usually induce a metabolic quiescence. Furthermore, nutritional limitation forces a growth quiescence (conservation of mass), and growth quiescence is an intrinsic part of size control in metazoans. How are these different aspects of quiescence related? They appear to form a causal hierarchy where metabolic quiescence can cause growth quiescence, and growth quiescence can cause cell cycle quiescence, but not the other way around. The causal hierarchy should be recognized in considering the mechanisms that cause quiescence.

Mass increase (growth) and cell number increase (proliferation) go hand-in-hand during the exponential growth of yeast culture or other cultured cells, and, among vertebrates, the bigger species have correspondingly more cells [11]. This has caused investigators to equate growth and proliferation. However, the coupling of growth and proliferation is frequently broken [7,34], and where it does exist, it is of interest to know whether the coupling is achieved because the cell cycle regulates growth, or growth regulates the cell cycle [34]. In an exponentially growing population of cells, mutations that specifically block cell cycle progress do not block continued increase in cell mass (although the rate of mass increase eventually switches from exponential to linear [35–37]). Reciprocally, when growth is arrested, usually by nutrient limitation, most cells arrest proliferation within one cell cycle of the arrest of growth [35–37]. Studies of this arrest suggest that cells have a size threshold below which they arrest cell cycle progress, usually in G1/G0 [35–37]. This size threshold imposes a unidirectional coupling of growth and cell cycle in which growth quiescence can enforce proliferation quiescence but not vice versa.

Considerations of the biology of growth and proliferation in metazoans also argue that cell cycle arrest is not sufficient for growth quiescence. This is apparent from the natural histories of many organisms, perhaps most notably the nematodes. The free-living nematode, *C. elegans*, grows to about 1 mm in length, whereas its parasitic cousins, such as *Ascaris*, reach about 400 times this length (about 10^7^ times the mass, roughly the magnitude of the fold difference between the size of a mouse and blue whale [7]). Despite the difference in size, *Ascaris* has the same anatomy and the same number of cells as the diminutive *C. elegans*. The size difference is achieved by continued postmitotic growth in *Ascaris*, illustrating that an arrest of cell proliferation does not terminate the growth phase. Experimental manipulation of the cell cycle in *Drosophila* demonstrated that cell cycle quiescence does not block growth [35–38]. For example, in proliferating imaginal disc cells, clonal loss of function of the essential cell cycle gene *string*, which encodes Cdc25, produced arrested cells that grew excessively large [35–37]. Thus, like the study of cultured cells, these analyses suggest that cell cycle quiescence is not sufficient to achieve growth quiescence.

Although less attention has been paid to the connection between metabolic quiescence and growth quiescence, growth is a major energy-consuming activity that cannot be maintained without metabolic activity, both anabolic activity to provide the material for growth and catabolic energy production to drive the process. But the opposite is not true. Growth quiescence does not cause metabolic quiescence. Two examples illustrate this. First, starvation of *E. coli* for glucose can stall growth, but it also induces the flagellar apparatus and active swimming. Second, the adult mammalian brain has ceased growing, yet is the seat of intense metabolic activity. Generally, unicellular organisms include metabolic quiescence as part of their response to nutritional deprivation, but in metazoans the onset of growth and cell cycle quiescence at maturity is usually not coupled to metabolic quiescence.

The consequence of the hierarchy in the regulation of different forms of quiescence is that different mechanisms give different forms of quiescence. Inhibition of cell cycle regulators suffices to give cell cycle quiescence but not growth quiescence, whereas inhibition of growth can give both cell cycle and growth quiescence. At first glance, the hierarchy suggests that one should not have situations in which growth is blocked but cells continue to divide. However, this can arise because a cell, such as an oocyte, that grows larger than the minimum threshold required for division can support continued divisions without growth until the size of the daughter cells drops below the size threshold for cell cycle progression. Finally, if metabolic quiescence were induced, then both growth and cell cycle quiescence would follow by indirect control.

## 8. Developmental programmes of quiescence

I am interested in focusing the present discussion on quiescence in the organism, as opposed to tissue culture models, because I believe that the latter give a distorted view of the relevant and predominant controls. Here, we focus on growth and proliferation, emphasizing a few examples from *Drosophila* in which genetic dissection defines the inputs.

Following fertilization and 13 extremely rapid and synchronous mitotic cycles that lack a G1 or G2 phase, a G2 phase is introduced in cell cycle 14. This pause in the cell cycle is created by elimination of maternally supplied mitotic activator, Cdc25 phosphatase, that removes inhibitory phosphates from cyclin:Cdk1 complexes. Most of the cells of the embryo only pause in cycle 14, because new expression of Cdc25 will drive cells into mitosis after a time delay that is proscribed by the position-specific cascade of developmental regulators [28,39]. However, a dorsally located tissue called the amnioserosa never re-expresses Cdc25 and never divides again. If a heat-shock-inducible Cdc25 (*string*) transgene is expressed during the G2 arrest, then the amnioserosa cells will be induced to divide [40]. As the embryo ages, the amnioserosa cells adopt a distinctive morphology, and become somewhat less responsive to Cdc25, but it is nonetheless clear that Cdc25 is initially the factor that limits the progress of the cell cycle [27,40]. Mutations affecting patterning of the embryo cause Cdc25 to be expressed in the region of the amnioserosa and lead to division [41]. Hence, these cells, which represent the earliest quiescent cells of embryogenesis, are arrested in G2 of cell cycle 14 because they lack Cdc25.

Most of the cells of the *Drosophila* embryo progress beyond cycle 14, and execute three more divisions that are driven by periodic pulses of Cdc25 gene expression. After mitosis 16, most of the cells pause in the first G1 phase [27,42,43]. Cell cycle regulation is substantially restructured to introduce this G1 quiescence in cycle 17. The expression of several cell cycle regulators changes during cycle 16, and three of the changes are required for the appearance of a G1 phase following mitosis 16: cyclin E expression is shut-off; expression of a cyclin E inhibitor, Dacapo, is activated; and an activator of the anaphase promoting complex (APC), Fizzy-related (Fzr), is expressed [42,44,45]. Shut-off of new cyclin E expression as well as Dacapo inhibition of persisting cyclin E : Cdk2 reduces this G1 cyclin function to levels below those required to initiate S phase following mitosis 16 [42,44–46]. Additionally, the expression of Fzr (a Cdh1 homolog) maintains the activity of the APC to promote postmitotic destruction of mitotic cyclin Cdk [45], which otherwise has a capacity to drive S phase [42,47]. In addition to these changes in gene expression, two other factors contribute to the emergence of G1 quiescence. E2F1, which was previously stable, is degraded during S phases 15 and 16, and its absence was shown to be important because induction of E2F1/Dp1 triggers S phase shortly after mitosis 16 [48,49]. The *Drosophila* retinoblastoma gene product, Rbf1, also plays a role in stabilizing the G1 following mitosis 16. If both maternal and zygotic sources of Rbf1 are eliminated, then mutant embryos exhibit ectopic E2F-dependent gene expression and S phase 17 after a transient G1 [50]. In addition to these genes, whose function influences the appearance or the stability of the G1, cycle 16 cells exhibit additional changes in cell cycle regulators such as an extinction of the expression of cyclin A, cyclin B and Cdk1 [51,52]. Altogether, these changes reveal a large-scale reconfiguration of cell cycle regulation in association with the introduction of G1 quiescence in cycle 17. Importantly, the changes in expression of various cell factors occur in a spatially programmed fashion and they continue even in an embryo whose cell cycle is arrested [42,43]. Thus, the changes that introduce G1 quiescence are programmed by developmental signals independent of the progress of the cell cycle.

The future fates of cells that arrest in G1 of cycle 17 during embryogenesis are varied. Most of the cells will never divide again, but will grow, enter programmed cycles of endoreduplication and will build the various larval tissues. Others will remain quiescent through the remainder of embryogenesis, re-enter the cell cycle during the first larval stage and will develop into the various discs. Here, I would like to highlight the fate of histoblasts, which ultimately form the epidermis of the adult abdomen.

Small clusters of histoblast cells are specified in each embryonic abdominal segment primordium. These enter proliferation quiescence after the 16 embryonic cycles, as mentioned already, and remain quiescent for the next few days, throughout the remainder of embryogenesis and all of larval growth. Although initially arrested in G1 of cycle 17, the histoblasts replicate their DNA and by early larval stages are in G2, where they remain arrested owing to failure to express Cdc25 [53]. Although in cell cycle quiescence, the histoblasts continue to grow during the long G2 arrest and they also accumulate cyclin E and probably other components required to advance cells from G1 into S phase [53]. Thus, at the end of the larval period, these cells are large and primed to proliferate, save for the lack of Cdc25. After pre-pupa formation, a rise in the ecdysterol hormone induces the expression of Cdc25 and activates a series of three rapid cell cycles (2.5 h) that lack a G1 phase. During these rapid cycles, the large histoblasts are reduced in size. These rapid cycles are followed by slower cycles (5–8 h doubling time) that are growth factor (Spitz and epidermal growth factor receptor) dependent. These cycles have a G1 phase, and cell duplication is accompanied by a doubling of cell mass so that cell size then remains constant. The insulin receptor and phosphatidyl inositol 3-kinase signalling are required for the growth of the histoblasts during these later divisions, and mutants in these pathways lead to an arrest of proliferation with small cells. The rapid proliferation of the histoblasts produces a population of cells which will form the abdominal epithelium of the adult. This example provides a nice case study that illustrates how a variety of controls can be brought into play to manipulate growth, proliferation and quiescence [53].

Analyses of proliferation histories of different tissues of the fly, while uncovering considerable diversity, also suggest some generalities. In contrast to views of the cell cycle as an autonomous oscillating system, at times cell cycle mechanisms abdicate responsibility for timing cell cycle progress to developmental cues that control expression of a cell cycle regulator (e.g. Cdc25) to dictate where and when cells will transit a step of the cell cycle [8]. Furthermore, even when considering the same population of cells, the mode of cell cycle regulation can change dramatically from one stage to another. In the example of the histoblasts, we have seen cycles controlled at the G2–M transition, cycles controlled at G1–S, cycles in which growth does not accompany division, cycles where growth and division are coupled, and we have seen that different cell regulators can act as the pivotal determinants of cell quiescence. Despite the intricate programmes of proliferation control, commonalities suggest that a limited number of pathways are deployed in various combinations to produce the varied programmes. Hopefully, we can identify the fundamental control circuits without losing sight of the how these programmes are deployed *in vivo*.

## 9. Are tumour suppressors the regulators of quiescence?

Cancer biology has proved to be a powerful context to identify mutations that disrupt quiescence and give rise to uncontrolled growth in humans, but the parallels are not simple. Activation of quiescent cells is only one of the changes required for sustained pathological growth [54], and even this one step appears extremely complex. Nonetheless, some tumour suppressor genes have pivotal roles in suppressing proliferation or growth. However, studies of the roles of these genes in normal biology have not really shown that these genes are essential for cellular quiescence, but this appears to be the consequence of inflated expectations rather than a lack of contribution to quiescence.

The retinoblastoma gene (Rb), the archetype of the tumour suppressor class of genes, has characteristics of a pivotal regulator of quiescence [55]. Rb and its relatives, the pocket proteins, interact with the key transcription factor, E2F, converting it from an activator of S phase genes to a suppressor of S phase genes. Distant relatives of Rb are found in organisms from yeast to humans, and in these organisms Rb has a similar interaction with a key S phase transcription factor [50,56,57]. Thus, Rb is a central and conserved regulator of cell cycle quiescence, and loss of this regulatory pathway appears to make a key contribution to the ‘success’ (rampant growth) of tumours. This has attracted a great deal of attention to the pathways that drive the dissociation of Rb from E2F, which are presented as fundamental to the control of cell cycle progress. Accordingly, a G1 cyclin:Cdk complex (cyclin D:Cdk4 or cyclin D:Cdk6) phosphorylates Rb, promotes its dissociation from E2F and activates expression of S phase functions particularly cyclin E. Cyclin E then functions in conjunction with Cdk2 to promote the S phase. While the steps described have been extensively documented, an analysis of *in vivo* phenotypes of mutations that disrupt these steps in mice, flies and worms suggests that this regulatory scheme is not globally required.

Knockout of Rb in mouse is lethal, but the homozygous embryo reaches foetal stages with a well-established body plan and differentiated tissues [58–60]. Additionally, if a chimera containing a mix of Rb mutant and normal cells is made, then the mouse is viable and Rb mutant cells contribute widely to the tissues of the adult [61]. These findings show that development, differentiation and stable quiescence occur in the absence of Rb function despite its proposed role in the fundamental regulatory switch stalling cells in G1.

One simple possible explanation for the dispensability of Rb is that related regulators, the ‘pocket proteins', might substitute for it. In this case, one would expect that G1–S regulation would still be intact in the Rb mutant. Significantly, an analysis of Rb mutant cells in brains of chimeric mice revealed that these cells had 4N DNA content [62]. This finding implies that the Rb mutation did disrupt the normal restriction in the progression of the cell cycle from G1 to S phase, but the cells nevertheless successfully differentiated as neurons and remained quiescent, but now quiescent in G2. Thus, the lack of a quiescence phenotype is not owing to redundant activities (e.g. the related pocket proteins) filling in for Rb, but rather appears to be owing to the action of alternative pathways that can mediate quiescence, at least in the affected neurons of the brain, despite the disruption of the Rb/E2F switch. Studies in model organisms support the idea of redundant pathways contributing to quiescence.

Studies of growth control in proliferating cells of the wing disc reveal a remarkable flexibility in the regulatory inputs into cell cycle progression and growth. Neufeld *et al*. [36] induced recombination that gave rise to clones of cells with altered activity of various cell cycle regulators within the wing disc epithelium. If they over-expressed the Cdc25 phosphatase encoded by the *string* gene in these clones, then G2 was shortened, but there was a compensatory increase in G1, and cell size remained roughly constant with the overall expansion of the clone (area occupied by the progeny cells) remaining unchanged. Reciprocally, shortening of G1 caused by increased expression of cyclin E resulted in a prolonged G2 and gave little change in growth. Disruption of the Rbf/E2F regulation affected both G1 and G2 regulation, but its consequences were still buffered by other controls. For example, over-expression of both subunits of the E2F transcription factor E2f1/Dp promoted the expression of both cyclin E and Cdc25; in the presence of an apoptosis inhibitor, this treatment promoted proliferation of the cells. However, the cells did not exhibit unregulated growth. The individual cells were smaller and overall expansion of the clone was unchanged. Reciprocally, over-expression of Rbf1 slowed proliferation of the cells, but now the cells were bigger and again there was little influence on the expansion of the clone. Apparently, growth control was not disrupted by changes in the cell cycle and this growth control had a homeostatic influence on the size of the clone; the cells comprising these clones ultimately entered quiescence after making an appropriate contribution to overall tissue growth. These findings show that even in a context in which the Rb pathway influences cell cycle progress, there are other pathways that have an impact on growth and eventually arrest proliferation of the cells.

As described already, wing disc cells arrest proliferation at least transiently once the disc reaches full size. The majority of the cells are arrested in G2 during this stage [22] in a Cdc25 limited state [63]. The cells remain arrested in G2 for about 12 h after initiation of the formation of the pupa and the beginning of wing morphogenesis. Then most cells go through two mitoses as they differentiate and arrest permanently in G1 about 12 h later [23,24]. In an effort to determine the role of Rb and associated regulators in this final quiescence, clones of cells were induced in which different cell cycle regulators were inactivated or over-expressed during the pupal stages [64,65]. The results suggested a progressive increase in the stability of quiescence in that several perturbations that inactivate Rb and/or activate E2F were able to extend cell cycle activity for one more cycle, but no more than one cycle. However, if measures were taken to deregulate additional steps, then cell cycles could be maintained through later stages. For example, over-expression of cyclin E and Cdc25 within a clone of cells lacking Rbf1 induced extensive additional cycles and overgrowth [64,65]. These findings suggest that the initial arrest of the cells in their terminal G1 phase relies on the Rb/E2F pathway, but that several barriers to proliferation arise as the developmental programme progresses.

The diverse programmes of control exhibited at different times and in different tissues create situations in which particular tumour suppressors can be crucial to the regulation of proliferation during distinct windows of time in particular lineages, whereas at other stages different pathways take regulatory responsibility. A recent study of the development of the optic lobe of *Drosophila* from a neuroepithelium provides a nice example of this [66]. The slowing of proliferation in the neuroepithelium precedes differentiation into neuroblasts, which then exhibit a distinct programme of divisions. The Hippo pathway is required to constrain proliferation in the neuroepithelium and for the developmental transition to neuroblasts. At this stage, activation of Yorkie, the transcription factor suppressed by the Hippo pathway, drives massive overgrowth. Apparently, this transformation-like phenotype results from the combined abilities of Yorkie activity to block the differentiation of the neuroepithelial cells and to drive the proliferation of these cells. After differentiation of the neuroepithelial cells into neuroblasts, the Hippo pathway is no longer required to suppress growth. Even full activation of Yorkie does not drive proliferation subsequent to neuroblast differentiation.

These *in vivo* analyses show that tumour suppressor genes make important, conserved and widespread contributions to cellular quiescence. However, they also illustrate the significance of backup and alternative modes of regulation that can cover for the loss of a tumour suppressor function at different times and places. In the context of these overlapping regulatory inputs into quiescence, it is not surprising that the consequences of tumour suppressor mutations are markedly tissue-specific.

## 10. The concept of growth factors as surrogates for nutrients

The discussion has emphasized the fact that cellular quiescence in metazoans is developmentally programmed, and connected to size control. Yet, no matter the complexity of the developmental inputs and multiple modes of cell cycle arrest, some commonalities exist in the cellular changes associated with the transition from active growth to quiescence. The most obvious common point is that actively growing cells make an abundance of the components that are needed for growth, and in widely diverse species the machinery for protein synthesis is one of the key contributors to growth [67–69]. As down-regulation of growth genes is common to the transition to quiescence, we might expect conservation of the cellular pathways underlying quiescence. However, an encompassing synthesis would have to account for the distinctions in the types of regulatory inputs in single-cell organisms and in metazoans.

Single-cell organisms respond to nutrient limitation with cell cycle quiescence, and often it is the nutrients themselves that provide the signal to enter quiescence. In contrast, in the more complex metazoans, homeostatic physiological controls provide cells with a consistently nutritive environment, and cells enter quiescence for other purposes as discussed already. Instead of nutrients, growth factors and cytokines provide the key regulatory inputs. This change might be made while preserving the cellular signalling mechanisms if the cellular pathways governing quiescence just switch their inputs. Thus, while a single-celled predecessor of metazoans might have sensed many nutritional signals to properly adjust its growth rate, a metazoan might sense growth factors and cytokines, each of which functions as a surrogate of a nutrient.

The concept of growth factors as surrogates of nutrients opens up the possibility that mechanisms of quiescence used in single-cell model organisms might be relevant to mammals. However, the concept is not as unifying as one might think. Even when evolution conserves regulatory logic, the circuitry of the regulation can differ [70,71], and clearly there have been changes in the cellular pathways that regulate the expression of the protein synthesis machinery. For example, ppGpp (magic spot), which is used as a specialized regulator of ribosomal RNA synthesis and activity of the translational apparatus in *E. coli* [72–74], has diverse uses among prokaryotes and is not found in eukaryotes [74]. Additionally, the regulators of growth genes in *Saccharomyces cerevisiae* are not conserved even among yeasts [71]. Of the various cellular regulators of growth, the target of rapamycin (TOR) pathway is perhaps the most conserved, at least in the sense that the TOR kinase appears to have important inputs into growth in *S. cerevisiae*, *Drosophila* and mammals, and the immediate regulators upstream and downstream of TOR regulation are conserved. However, the TOR regulatory module appears to be used in growth regulatory circuits that have diverged significantly. For instance, comparison of nutrient regulation of TOR in *S. cerevisiae* with growth factor inputs in flies and mammals shows differences well beyond a simple substitution where a growth factor plays a role analogous to the nutrients. The major inputs into the TOR pathway in metazoans are mediated by tyrosine kinase receptors, a class of proteins not found in yeast. Thus, major modifications of signal transduction distinguish the upstream and downstream steps in the action of TOR in growth control.

In summary, the biology of quiescence reveals diverse regulatory inputs. There are notable parallels in the regulation of quiescence between *Drosophila* and mammals. However, within both mammals and flies there are diverse modes of regulating quiescence. The parallels suggest that a plurality of mechanisms may be conserved among metazoans, but it remains to be seen whether the manner of deployment of these various mechanisms is also conserved. When one looks beyond metazoans, there are examples of regulatory diversification indicating that pathways regulating quiescence have been profoundly modified during evolution.
